# Case report: A case of acute pancreatitis with myocarditis and thyrotoxicosis

**DOI:** 10.3389/fcvm.2025.1527970

**Published:** 2025-06-30

**Authors:** Qiuyu Fu, Jianing Cui, Yanli Zhang, Jiamian Liu, Xiaoqiang Zhang

**Affiliations:** ^1^ Department of Vasculocardiology, Chengdu Second People’s Hospital, Chengdu, Sichuan, China; ^2^ School of Medicine, University of Electronic Science and Technology of China, Chengdu, China; ^3^ Organ Transplant Center, Sichuan Provincial People's Hospital, University of Electronic Science and Technology of China, Chengdu, China; ^4^ Department of Pediatrics, West China Second University Hospital, Sichuan University, Chengdu, Sichuan, China

**Keywords:** acute pancreatitis, myocarditis, thyrotoxicosis, tomography scan, case report

## Abstract

**Background:**

A variety of diseases can complicate acute pancreatitis and myocarditis. As acute pancreatitis combined with myocarditis and thyrotoxicosis is rare, we report a case of acute pancreatitis combined with myocarditis and thyrotoxicosis.

**Case presentation:**

We present a case of a previously healthy 37-year-old male who was admitted with epigastric pain; elevated blood amylase, lipase, and cardiac enzymes; markedly abnormal thyroid function; and an electrocardiogram showing elevated ST segments. He was diagnosed with acute pancreatitis combined with myocarditis and thyrotoxicosis. After 10 days of treatment, his epigastric pain disappeared. Further, his clinical test indices and electrocardiogram results returned to normal. The patient was free of abnormalities at the one-month follow-up, and his prognosis was good.

**Conclusion:**

Acute pancreatitis can be complicated by myocarditis and thyrotoxicosis. The aetiology and pathogenesis are unknown and warrant further study.

## Introduction

Acute pancreatitis is a common acute abdominal disease that is complex and variable. The degree of severity varies, and mild cases can manifest as pancreatic oedema, which is common and often self-limiting, so the prognosis is good (1). Myocarditis refers to myocardial inflammation with multiple clinical manifestations; viral myocarditis is not uncommon. The complications of acute pancreatitis and myocarditis caused by various pathogens, such as Coxsackie virus, have been previously reported. Takizawa et al. (2–5) reported four cases of adult patients with myocarditis and pancreatitis caused by Coxsackie virus infection involving serotypes A4, B1, B2, B4, and B5. Thyrotoxicosis may lead to the development of acute myocarditis (6). Acute pancreatitis complicating thyrotoxicosis is rarely reported. We were prompted to present this case because the patient presented with concurrent acute pancreatitis, myocarditis, and thyrotoxicosis.

## Case presentation

**Figure 1:**
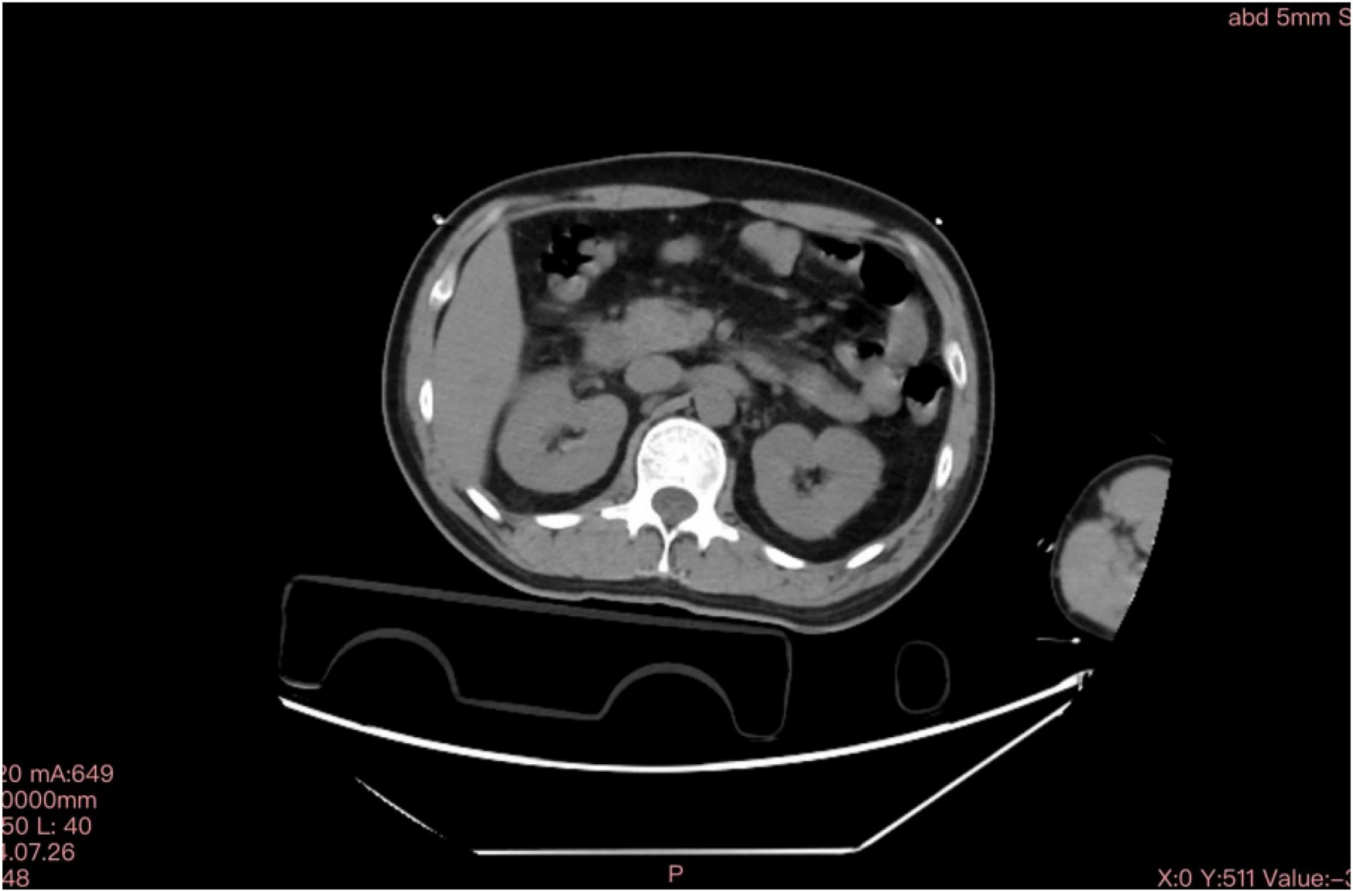
Abdominal computed tomography scan on July 26,2024. Swollen pancreas without gallbladder stones.

**Figure 2:**
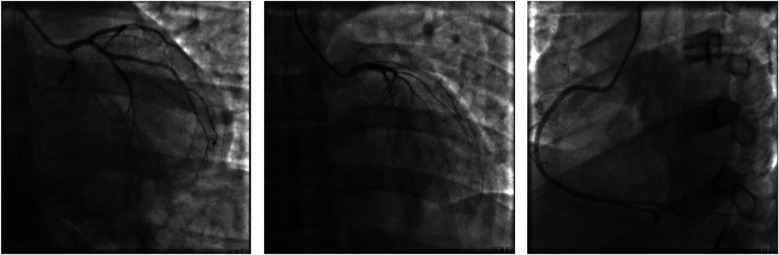
Coronary angiogram on July 26, 2024. No significant thrombi or stenosis in the lumina of the left and right coronary arteries, with anterograde flow achieving TIMI grade 3.

**Figure 3:**
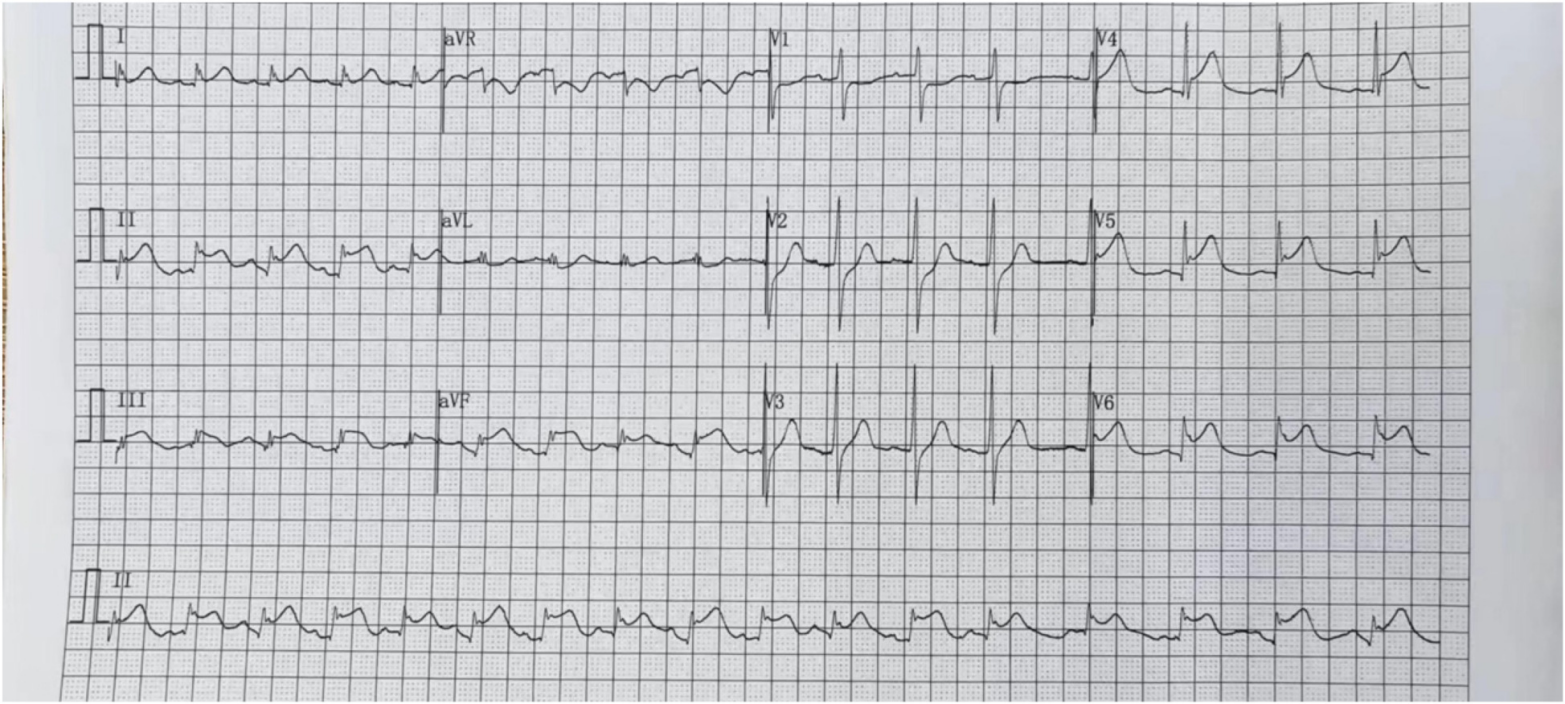
Electrocardiogramon July 26, 2024. ST-segment elevation in leads II, III, AVF, V4–V6.

**Figure 4:**
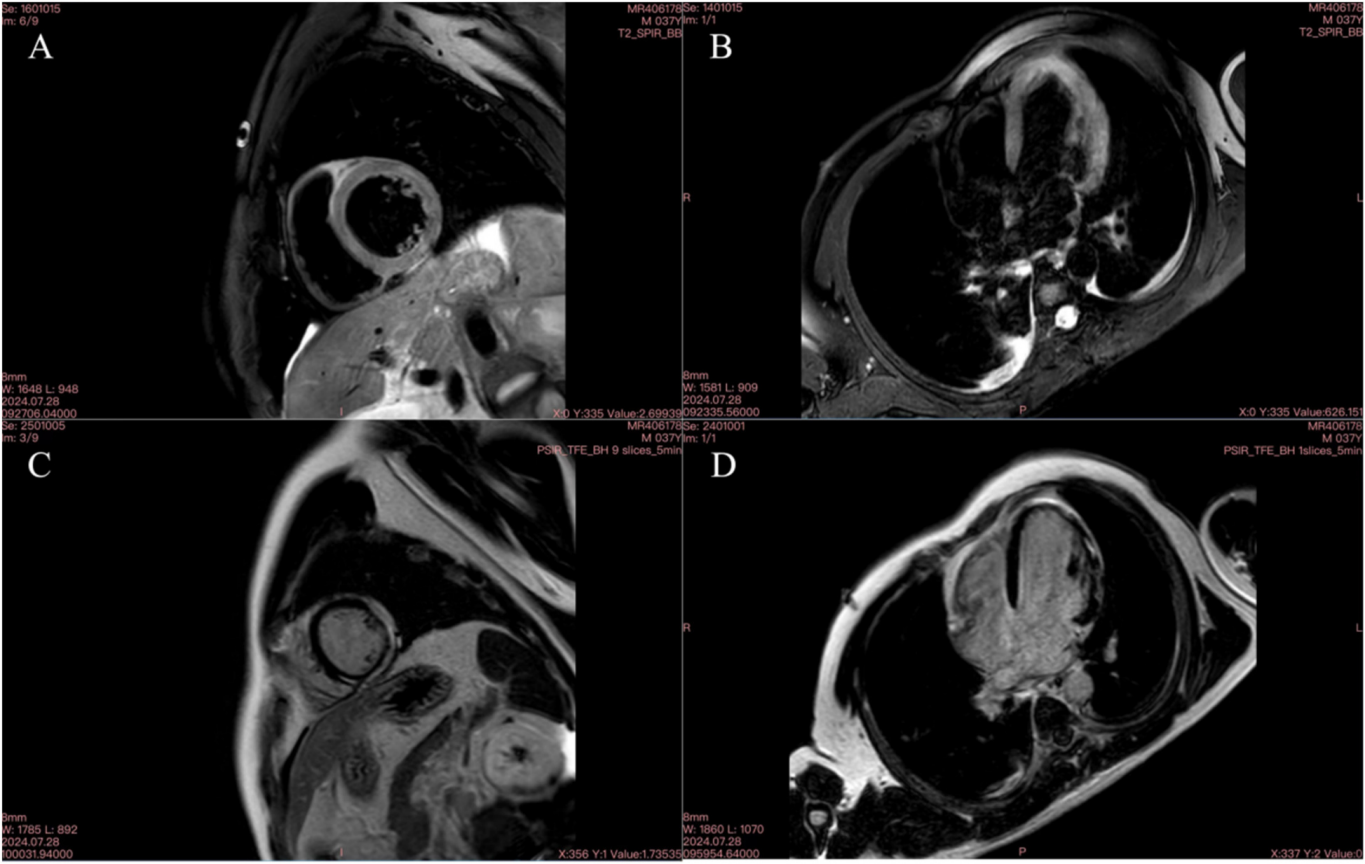
Cardiac MR image on September 28, 2024. **(A)** T2 fat-suppressed image of the left ventricular short axis showing multiple oedemas; **(B)** T2 fat-suppressed image of the four-chambered heart showing multiple oedemas; C delayed enhancement of a small amount of the subepicardial myocardium in the lateral wall of the short axis of the left ventricle; D delayed enhancement of a small amount of the subepicardial myocardium in the lateral wall of the four-chambered heart.

**Figure 5:**
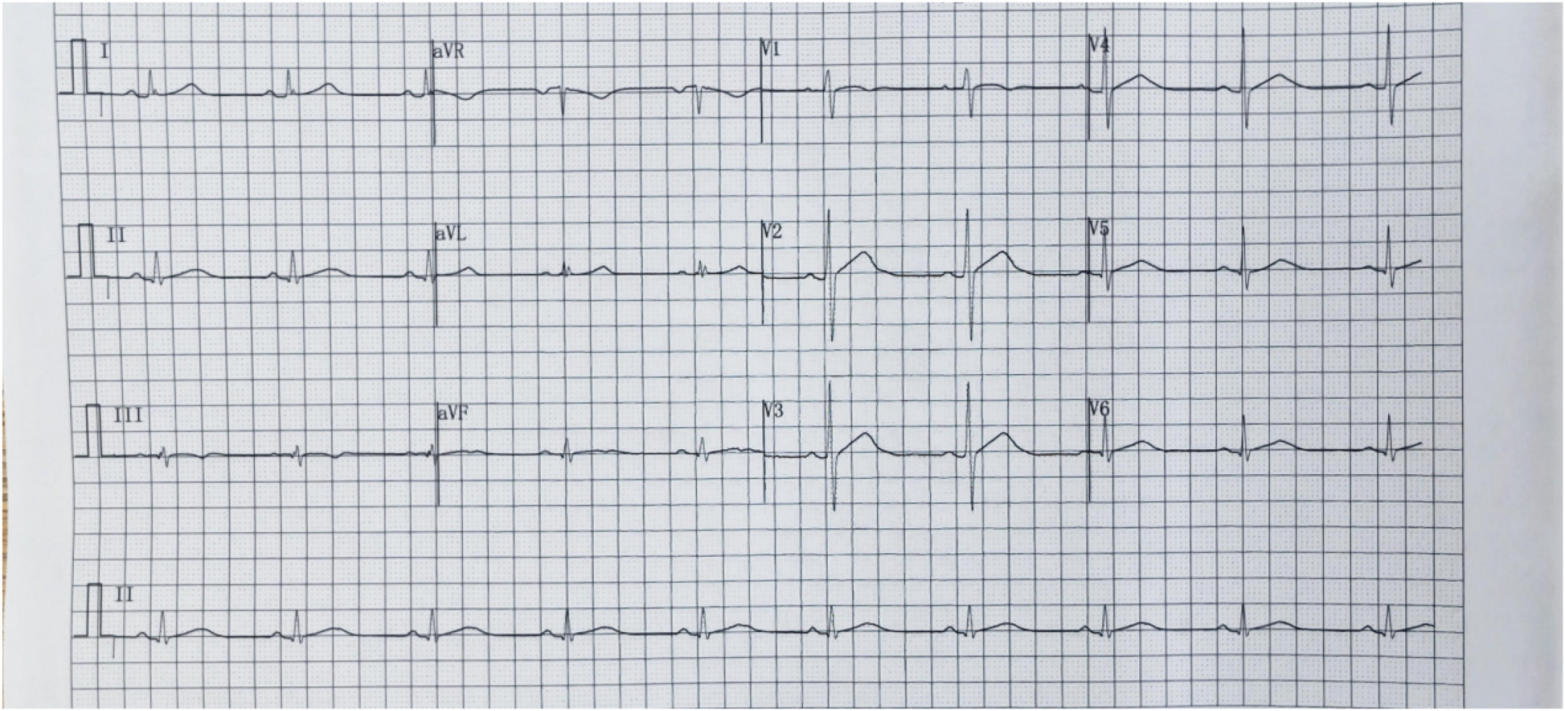
Electrocardiogram on August 2, 2024. Elevated ST segment return to normal.

We report a case of a 37-year-old male who was admitted to a local hospital with epigastric pain and fever. Acute pancreatitis was initially diagnosed on the basis of an abdominal computed tomography scan suggesting pancreatic swelling (Figure 1) and elevated blood amylase. He was referred to our hospital after completing an electrocardiogram with ST-segment elevation in the inferior wall leads, a coronary angiogram with no abnormalities (Figure 2), and a significant abnormality in thyroid function. He was in good health and had no history of alcohol abuse. At the time of admission, his axillary temperature was 37.2°C, his heart rate was 117 beats/min, his respiratory rate was 22 breaths/min, and his blood pressure was 116/64 mmHg. Thyroid examination showed no enlargement or pressure pain. No murmur was heard on cardiac examination. The abdominal muscles were not tense, and there was pressure in the epigastrium without rebound pain. Subsequent tests revealed that the following were all significantly higher than normal: blood amylase, 206 U/L (normal range, 35–135 U/L); lipase, 291 U/L (normal range, 5.6–51.3 U/L); cardiac enzymes, ultrasensitive troponin T, 3,023 ng/L (normal range, <14 ng/L); creatine kinase CK, 1,365 U/L (normal range, 15–163 U/L); acid kinase isoenzyme CKMB, 88.30 ng/ml (normal range, 0–4.87 ng/ml); ultrasensitive C-reactive protein hsCRP, 95.86 ng/L (normal range, 0–5 ng/L); and interleukin, 6–31.4 Pg/ml (normal range, <7 Pg/ml). Moreover, the following were markedly abnormal: thyroid function, T3, 3.53 pmol/L (normal range, 0.92–2.79 Fpmol/L); T4, 194.10 pmol/L (normal range, 58.10–140.60 pmol/L); FT3, 11.78 pmol/L (normal range, 3.5–6.5 pmol/L); FT4, 40.52 pmol/L (normal range, 11.; and TSH: 0.014 uIU/ml (normal range 0.55–4.78 uIU/ml). In addition, his triglycerides (1.88 mmol/L; normal range <1.7 mmol/L) were mildly elevated. The patient's thyroid peroxidase antibody (TPO-Ab) level was <28.00 IU/ml (0–60.00) and thyrotropin receptor antibody (TRAb) level was <0.80 IU/L (0–1.75). His white blood counts, calcitoninogen, and glucose were within normal limits, and the polymerase chain reaction (PCR) results were negative for several viruses (adenovirus, rhinovirus, respiratory syncytial virus, coronavirus, Coxsackie virus, influenza virus, cytomegalovirus, toxoplasma gondii, herpes virus, and others) as well as Mycoplasma pneumoniae. An electrocardiogram revealed sinus rhythm with q waves visible in leads II, III, and AVF and ST-segment elevation of 0.1 mv and T-wave inversion in leads II, III, AVF, and V4–V6 (Figure 3). Contrast-enhanced cardiac magnetic resonance imaging showed slightly oedematous and scattered delayed subepicardial enhancement of the left ventricular myocardium, which was slightly pronounced in the lateral wall of the left ventricular midsection (Figure 4). A repeat abdominal computed tomography scan suggested pancreatic swelling without gallbladder stones. Cardiac ultrasound showed no significant abnormalities, and the left ventricular ejection fraction was 54%. Thyroid ultrasound revealed that the thyroid parenchyma was echogenic and homogeneous, no clear occupancy was observed, and no abnormal blood flow signal was observed.The patient was diagnosed with acute pancreatitis complicated by myocarditis and thyrotoxicosis. He was treated with bowel rest, nutritional support, growth inhibitors, trimetazidine, coenzyme Q10, and propranolol. One week later, his temperature had normalized, and his abdominal pain had resolved. After 10 days, his amylase, lipase, and ultrasensitive C-reactive protein levels were lower than before. In addition, his levels of cardiac enzymes and thyroid function returned to normal. The electrocardiogram was normalized (Figure 5). After one month of follow-up, the patient did not complain of discomfort, and the repeat abdominal CT revealed no abnormalities. Further, his amylase and cardiac enzyme levels as well as his thyroid function were normal.

## Discussion

Common causative risk factors for acute pancreatitis include biliary tract disease, alcohol consumption, and metabolic diseases, whereas other pathogenic factors include poor diet, infection, and autoimmune diseases (7). The patient we reported on had no previous history of biliary tract disease, alcohol consumption, drug use, or endocrine or immune system disease as well as no abnormalities on complete pathogenetic examination. Moreover, the aetiology of the disease was unclear, and it was induced by drinking cold beverages. The patient presented with fever, abdominal pain, and elevated blood amylase and lipase levels. An abdominal computed tomography scan revealed that his pancreas was enlarged, which could be used to diagnose acute pancreatitis. After symptomatic and comprehensive treatment was provided, the patients' condition improved significantly. After presenting with concurrent acute pancreatitis, myocarditis, and thyrotoxicosis, and the patient had a favourable prognosis.

The clinical manifestations of myocarditis range from mild symptoms, such as chest pain, fever, sweating, chills, and dyspnoea, to more severe manifestations, such as palpitations, syncope, or sudden cardiac death due to ventricular arrhythmias or atrioventricular block (8). In this case, the patient had no obvious cardiac symptoms or signs. His cardiac enzymes were significantly elevated, electrocardiogram showed ST-segment elevation, coronary arterial vascularization was normal, contrast-enhanced cardiac magnetic resonance imaging revealed slight oedema of the left ventricular muscle and scattered subepicardial delayed enhancement, and left ventricular intermediate part of the lateral wall was slightly obvious, which aligns with the diagnostic criteria for myocarditis (9). Consequently, a diagnosis of acute myocarditis was made. The patient had no typical cardiac manifestations, which made his condition easy to misdiagnose, and the electrocardiogram showed ST-segment elevation, which could have led to a misdiagnosis of another cardiac disease and should be taken seriously.

Myocarditis can be attributed to infectious agents, drugs and poisons, and immune-mediated mechanisms. Viral myocarditis is the most common. Other infectious causative agent associated with acute myocarditis and pancreatitis include leptospirosis, hepatitis E, enterovirus, West Nile virus, EBV, and mumps, except for coxsackievirus (10–17). Jiang GM, et al., reported concurrent pancreatitis and cardiomyositis in children with mesenchymal lupus erythematosus (18). Egashira F, et al. Cases of fulminant type 1 diabetes mellitus with pancreatitis and myocarditis (19). Although we screened for the causative agent and excluded common viruses by PCR, unfortunately we were not able to exclude all viruses.

In addition to common viruses, unfortunately, we could not rule out all viruses. Thyrotoxicosis may lead to acute myocarditis. Previously, Sen et al. reported cases of acute myocarditis associated with Graves' disease (6). Kwak et al. reported a case of thyrotoxicosis due to thyroid hormone abuse in a bodybuilder combined with Coxsackie virus B4 infection complicating acute myocarditis (20). This patient had markedly abnormal thyroid function and was admitted with an increased heart rate of 114 beats/minute, without other hypermetabolic manifestations, such as palpitations, tremors, excessive sweating, or emaciation, no enlargement of the thyroid gland, and negative associated antibodies. The endocrinology team consulted and considered thyrotoxicosis and used only propranolol and no antithyroid medication. Prior to discharge, the patient's thyroid function normalized. The cause of the thyrotoxicosis in this patient is currently unknown.

The relationship between acute pancreatitis and changes in thyroid function has also been studied. De Sola et al. (21) analysed changes in thyroid function in patients with acute pancreatitis and reported that 20% of them had low levels of T3 and that their thyrotropin levels were consistently within the reference range. In an 8-year cohort study of patients with acute pancreatitis with nonthyroidal illness syndrome (NTIS), QuC et al. (22) reported that 183 patients with acute pancreatitis were diagnosed with NTIS, with a prevalence rate of 64.7%. NTIS is commonly observed in patients with acute pancreatitis within seven days of the onset of the disease, and FT3 should be investigated as a potential biomarker for predicting death in patients with acute pancreatitis. Wang et al. (23) found that FT3, FT4, and TSH levels decreased with increasing severity of acute pancreatitis and FT3 may be a useful biomarker in the early assessment of acute pancreatitis severity. Unlike in previous studies, this patient presented with thyrotoxicosis and hyperfunction and did not show any decrease in T3 or FT3 during the course of the disease. With elevated T3 and FT3 levels, the patient had a good prognosis, which is consistent with the findings of previous studies.

Research has found that molecular events within pancreatic parenchymal cells and the regulation of immune cells are the main pathogenesis of acute pancreatitis (24). The pathogenesis of myocarditis involves the interaction between stimulating factors and the subsequent host immune response. Infectious causes, especially cardiotropic viruses, are the most common predisposing factors. However, autoimmune processes independent of microbial triggers, as well as toxic myocardial damage caused by drugs, chemicals or metabolic disorders, also lead to the development of myocarditis through multiple mechanisms (9). Immune—mediation is also an important pathogenesis of myocarditis (25). Autoimmune factors are important etiological factors for thyrotoxicosis (26). We speculate that this patient has concurrent acute pancreatitis, myocarditis and thyrotoxicosis, which may be related to inflammatory responses and immune—mediation and need further verification.

## Conclusion

Acute pancreatitis may be complicated by myocarditis, while concurrent thyrotoxicosis is rare. In this case, the etiology of pancreatitis remains undetermined, and the co-occurrence of myocarditis and thyrotoxicosis may be associated with inflammatory responses and immune-mediated mechanisms, worthing further investigation.

## Data Availability

The original contributions presented in the study are included in the article/Supplementary Material, further inquiries can be directed to the corresponding author.
